# A Model Partnership: Mentoring Underrepresented Students in Medicine (URiM) in Emergency Medicine

**DOI:** 10.5811/westjem.2020.9.48923

**Published:** 2020-12-07

**Authors:** Jae Goines, Elizabeth Iledare, Douglas Ander, Joshua Wallenstein, Ngozi Anachebe, Martha Elks, Nicole Franks, Melissa White, Philip Shayne, Megan Henn, Sheryl L. Heron

**Affiliations:** * Emory University School of Medicine, Department of Emergency Medicine, Atlanta, Georgia; † Morehouse School of Medicine, Department of Obstetrics and Gynecology, Atlanta, Georgia; ‡ Morehouse School of Medicine, Department of Internal Medicine, Atlanta, Georgia

## Abstract

**Introduction:**

Creating a racially and ethnically diverse workforce remains a challenge for medical specialties, including emergency medicine (EM). One area to examine is a partnership between a predominantly white institution (PWI) with a historically black college and university (HBCU) to determine whether this partnership would increase the number of underrepresented in medicine (URiM) in EM who are from a HBCU.

**Methods:**

Twenty years ago Emory Department of Emergency Medicine began its collaboration with Morehouse School of Medicine (MSM) to provide guidance to MSM students who were interested in EM. Since its inception, our engagement and intervention has evolved over time to include mentorship and guidance from the EM clerkship director, program director, and key faculty.

**Results:**

Since the beginning of the MSM-Emory EM partnership, 115 MSM students have completed an EM clerkship at Emory. Seventy-two of those students (62.6%) have successfully matched into an EM residency program. Of those who matched into EM, 22 (32%) have joined the Emory EM residency program with the remaining 50 students matching at 40 other EM programs across the nation.

**Conclusion:**

Based on our experience and outcomes with the Emory-MSM partnership, we are confident that a partnership with an HBCU school without an EM residency should be considered by residency programs to increase the number of URiM students in EM, which could perhaps translate to other specialties.

## INTRODUCTION

Creating a racially and ethnically diverse workforce remains a challenge for medical specialties, including emergency medicine (EM).^1^–^3^ In 2008 a set of recommendations designed to augment physician diversity in EM was published;^4^ however, a recent study suggested that these best practices have not been widely implemented.^5^ The pilot intervention in this study included three strategies focusing on a scholarship-based externship, a funded second-look event, and increased involvement of underrepresented in medicine (URiM) faculty in the interview and recruitment process. In response to these findings, a workgroup of emergency physicians with a focus on and expertise in diversity and inclusion reconvened in 2018. The workgroup identified several strategies to recruit diverse applicants into EM. Among the most commonly discussed strategies was visiting elective clerkships for URiM students. These programs have proliferated in the last decade, with over 30 such programs in EM identified in 2018. However, as of this writing there has been limited data to suggest that the extent of impact of these URiM dedicated programs.^6^

The challenge faced by EM residency directors in recruiting diverse applicants is in large part a reflection of the number of URiM within undergraduate medical education (UME).^7^ In a recent survey, 35% of program directors reported that the small pool of URiM applicants was the greatest barrier to recruiting a diverse class of residents.^5^,^6^,^8^,^9^ However, there are a small number of medical schools with a higher representation of URiM students; most prominent of these are the historically black colleges and universities (HBCU). Based on a recent Association of American Medical Colleges (AAMC) data set, there are only three HBCU medical schools: Howard University College of Medicine; Meharry School of Medicine; and Morehouse School of Medicine.

These institutions represent 2.4% of United States medical schools yet have 14% of all Black medical students in the US.^10^ Further, the mission and culture of these institutions generally emphasize care for the underserved and other principles of equity and justice that are considered to be core values of EM. The challenge, however, is that none of these institutions has an academic EM department or EM residency training program. This poses a significant barrier in recruiting students at HBCU medical schools who are interested in EM. In addition to not having easy access to EM advisors or mentors during the critical stages of the application process, students at HBCU medical schools may not have the same exposure to EM during the foundational early years of medical school, when a student’s choice of residency training/specialty is often considered and in some cases, solidified.

Morehouse School of Medicine (MSM), a HBCU school in the city of Atlanta, does not have an academic EM department or residency training program and has more students that are URiM than the average US medical school. Founded in 1975, MSM has a student population that currently identifies as approximately 70% URiM. Another medical school in Atlanta, Emory University School of Medicine, has a large academic EM department and residency training program. Emory EM has had a nationally recognized history of supporting diversity and inclusion in EM with key URiM faculty publishing one of the first papers on how to improve diversity in EMt.^11^ For the past 20 years there has been a meaningful partnership between MSM and the Emory Department of Emergency Medicine. Begun initially as an informal interaction, over the course of 20 years this relationship has flourished and become a more structured partnership that has resulted in significant outcomes with regard to matching HBCU students into EM residencies around the country.

## METHODS

In 1999 the Emory Department of Emergency Medicine began its collaboration with MSM to provide guidance to MSM students who were interested in EM. At the onset of this collaboration, it was primarily the EM clerkship director who provided the bulk of mentorship and group activities. Since then, the program has expanded to include engagement of more Emory EM faculty and residents. Every MSM student who expresses interest in EM can meet with the faculty leadership in Emory EM. This includes the clerkship director, assistant clerkship director, program director, and associate/assistant program director, as well as other EM faculty. In addition, there continues to be an increased effort to engage UriM faculty as mentors and role models for URiM students. These relationships incorporate shadowing opportunities, assistance with career decisions, guidance for planning away rotations, fourth-year scheduling, application assistance, interview guidance, and when requested by the student, help with their rank list decisions.

**Population Health Research Capsule**

What do we already know about this issue?

*Emergency medicine (EM) continues to have difficulty creating a racially and ethnically diverse workforce*.

What was the research question?

Does a structured partnership between an EM residency and a historically black college and university (HBCU) medical school result in more underrepresented medical students in EM?

What was the major finding of the study?

*A partnership between an EM residency and a HBCU medical school can help to increase the number of underrepresented in the field of EM*.

How does this improve population health?

*Partnerships like this can help to improve patient health outcomes, address healthcare disparities, and advance health equity*.

In addition, an Emory EM faculty member serves as the faculty advisor for the MSM EM interest group, and the interest group is also assigned a senior Emory EM resident liaison. One key component of this partnership is the equitable treatment within Emory EM of MSM and Emory medical students regarding opportunities and exposure. This includes the guarantee that MSM students, like Emory students, are given priority to the Emory EM rotation in high-yield months. To evaluate the proportion of matches before and after the partnership, we submitted data to a regression using a beta distribution and a logit link. The change following 1999 was analyzed using a linear spline with a single knot at 1999. We conducted analyses using R v 3.5.1, R Core Team (R Foundation for Statistical Computing, Vienna, Austria)

## RESULTS

MSM had its first student match into EM in 1985. Since the beginning of the MSM-Emory EM partnership in 1999, 115 MSM students have completed an EM clerkship at Emory. Seventy-two of those students (62.6%) have successfully matched into an EM residency program. Of those who matched into EM, 22 (32%) have joined the Emory EM residency program with the remaining 50 students matching at 40 other EM programs across the nation. To compare the proportion of MSM students who matched into an EM residency before vs after the MSM-Emory EM partnership, we conducted a linear spline in a beta regression. The significance level was assigned to alpha of 0.05. The spline in the regression was significant (odds ratio [1.10], 95% confidence interval, 1.01 – 1.20, *P* = .03). This finding indicates that the proportion of EM matches began to increase following the 1999 partnership.

MSM has undergone a period of rapid expansion in terms of class size. To control for this expansion, we also assessed the MSM-Emory EM partnership with regard to the percentage of total MSM students who matched into EM each year. Before the partnership, the average percent of the total MSM class matching into EM was 3.01%. Since the inception of the partnership, the total percent of the class matching into EM is 6.65%, which represents an increase of 121.20%. We performed descriptive analyses to further assess the match outcomes of the partnership as it progressed. Specifically, in the last six years (2012–2018), the mean candidates matching per year increased from 2.07 to 5.67. In the most recent two years of the partnership to date (2017–2018), the average number of EM matches was 9.0, which represented 15.79% and 11.84% of the total MSM senior class, respectively. This two-year period coincided with the period during which an Emory EM faculty member became adjunct faculty at MSM, and the EM rotation was certified as a senior elective, thus allowing this rotation to count toward required graduation credits. Further, over the last 10 years, MSM has matched at least one student to EM every year, which represents a notable increase over the 10 years prior.

## DISCUSSION

Over the past two decades, a successful partnership has developed and matured between the Emory Department of Emergency Medicine and MSM, resulting in a significant increase in the number of MSM students matching into EM. An increase in the diversity of residents and emergency physicians has been a goal for the Academy for Diversity and Inclusion in the Society for Academic Emergency Medicine, the premier organization for academic EM.^12^ Emergency physicians are at the forefront of patient care, and increasing the number of emergency physicians to align with the population we serve is a desired goal.^13^–^15^ For example, in 2017 there were 83,968 residents in US and Canadian allopathic medical schools, of whom only 13.67% classified as URiM.^16^ During the same period, out of 7136 EM residents only 4.42% of these identified as Black. This is clearly a significant disparity given that in the 2017 Census, 13.4% of the US population identified as Black.^17^

This level of disparity is especially problematic in a racially diverse city such as Atlanta, since previous literature consistently indicates that patient outcomes and satisfaction are improved when a patient and his or her physician share a racial and/or ethnic background.^6^,^13^,^18^–^20^ From its inception the MSM-Emory EM partnership has been intentional about closing this gap and has seen positive results. Since the partnership began, MSM has seen over twice as many students matched into EM as a percent of the overall class. Our analyses revealed that this was a significant difference, suggesting that the partnership has had a positive impact. The number of MSM students matching into EM has continued to rise as the MSM-Emory EM partnership grew and became more formalized.

In addition to continuing mentorship, application preparation, and previously noted activities, in 2017 an Emory emergency physician, who was the MSM EM faculty advisor, officially became adjunct faculty at MSM. Also, in that same year, MSM certified the Emory EM subinternship/clerkship as a senior selective, thus ensuring that it became part of the core curriculum for EM-bound and/or interested students who elected to take the rotation. Notably, in this same year, 15.79% of the MSM graduating class matched into EM, which is above the national average of the percent of US medical graduates matching into EM that year (9.16%).^21^

In our study there were 43 students enrolled in our EM elective who did not match in EM. We do not have details regarding their confidential rank list order or subsequent career choices. This missing data only strengthens our conclusion that our partnership was successful since we likely underestimated the number of students impacted by our mentorship who either ranked EM but matched in other specialties, or within several years were able to switch into EM.

This type of partnership requires a culture, climate, and commitment in diversity and inclusion for all students who have an interest in EM. Based on the success of the MSM-Emory EM partnership, we propose a multilayered approach to successful matching in EM residency programs for HBCU schools without an EM residency. Mentorship has been demonstrated to be a significant factor in URiM applicants being able to identify with a residency program; as such, mentorship has been maintained as the cornerstone of this partnership.^8^ Specifically, key aspects of this partnership include a dedicated group of Emory EM faculty and staff who provide the needed advising, mentoring, administrative support, and teaching throughout the MSM student’s years in medical school. From the perspective of a non-HBCU medical school, partnering with HBCU schools without a residency program in EM is an opportunity to increase URiM in EM and advance diversity and inclusion in the field.

Initiatives to increase URiMs in EM, including a paid elective, a funded second look and URiM involvement in recruitment, focus on later stages of a student’s decision-making process. Our focus is on a close relationship between an academic EM department and URiM students from a partner school to nurture an early interest in EM. As this relationship, mentoring, and advising continues throughout their medical education the opportunity of matching in EM is improved. Our holistic approach to EM mentorship for the MSM students has resulted in positive match outcomes exceeding national norms. We hope that the MSM-Emory EM partnership can serve as a model for other residency programs that value diversity and desire to increase the diversity of their residency classes in other specialties in medicine.

## LIMITATIONS AND FUTURE DIRECTIONS

The program we describe has some identified areas for growth. First, although we discussed the many benefits of increasing URiMs in EM residency programs, we do not specifically describe the racial or ethnic demographics of the MSM students matching into EM. Over the course of the Emory-MSM relationship, specific racial and ethnic data was not collected, and doing so retrospectively would have been complex. It should be noted that although on average 70% of MSM students identify as URiM, MSM also accepts and trains non-URiM students who have benefitted from the Emory-MSM partnership. Future analyses on this partnership would benefit from additional information specific to race/ethnicity and also socioeconomic status.

Additionally, as this is a single institutional program, the statistics are limited by sample size and meant to be descriptive only. With the increasing numbers of students contemplating EM as a career and ultimately applying for residency, as well as the increasing complexity of the application process, we recognized the need to better formalize our partnership and provide a structure for interested medical students throughout all years of medical school. In the last two years, for example, structured “EM bootcamp” sessions which include simulations, application discussion, and rotation preparation, have been instituted prior to the start of fourth-year subinternship rotations.

## CONCLUSION

The Emory-MSM partnership has shown success in increasing the presence of underrespresented students in EM with targeted intervention/involvement/efforts in the early stages of a medical student’s specialty selection process. This type of program, which mentors URiM students early and engages them in multiple aspects of the specialty and its application process, should be adopted by other HBCU medical schools and all US medical schools. Given the limited published data, previous attempts to increase URiM student interest in EM, while likely beneficial, were less effective than our broad-based approach, or at this juncture, are unknown. Based on our 20-year experience with the Emory-MSM partnership and our outcomes, we are confident that our approach is effective and that partnership with an HBCU school should be considered by residency programs to increase the number of URiM students in residency programs.

## Figures and Tables

**Figure:**
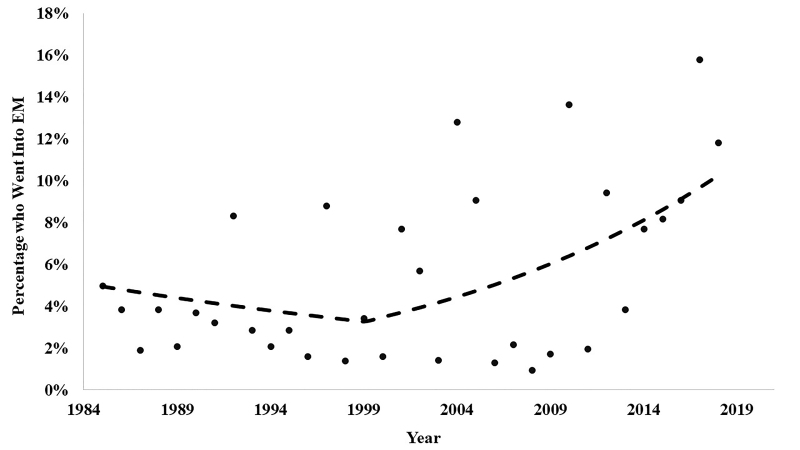
Percentage of Morehouse School of Medicine students matching into emergency medicine 1984–2018.

## References

[1] Landry AM, Stevens J, Kelly SP (2013). Under-represented minorities in emergency medicine. J Emerg Med.

[2] Martin ML (2000). The value of diversity in academic emergency medicine. Acad Emerg Med.

[3] Grumbach K, Mendoza R (2008). Disparities in human resources: addressing the lack of diversity in the health professions. Health Aff (Millwood).

[4] Heron SL, Lovell EO, Wang E (2009). Promoting diversity in emergency medicine: summary recommendations from the 2008 Council of Emergency Medicine Residency Directors (CORD) Academic Assembly Diversity Workgroup. Acad Emerg Med.

[5] Boatright D, Tunson J, Caruso E (2016). The impact of the 2008 Council of Emergency Residency Directors (CORD) Panel on Emergency Medicine Resident Diversity. J Emerg Med.

[6] Garrick JF, Perez B, Anaebere TC (2019). The diversity snowball effect: the quest to increase diversity in emergency medicine: a case study of Highland’s emergency medicine residency program. Ann Emerg Med.

[7] Lett LA, Murdock HM, Orji WU (2019). Trends in racial/ethnic representation among US medical students. JAMA Netw Open.

[8] Association of American Medical Colleges (2015). Altering the course: Black males in medicine.

[9] Figueroa O (2014). The significance of recruiting underrepresented minorities in medicine: an examination of the need for effective approaches used in admissions by higher education institutions. Med Educ Online.

[10] Rodríguez JE, López IA, Campbell KM (2017). The role of historically black college and university medical schools in academic medicine. J Health Care Poor Underserved.

[11] Heron S, Haley L (2001). Diversity in emergency medicine--a model program. Acad Emerg Med.

[12] Society for Academic Emergency Medicine Academy for Diversity and Inclusion in Emergency Medicine: Mission Statement and Objectives.

[13] Betancourt JR, Maina AW (2004). The Institute of Medicine report “Unequal Treatment”: implications for academic health centers. Mt Sinai J Med.

[14] Nelson AR (2003). Unequal treatment: report of the Institute of Medicine on racial and ethnic disparities in healthcare. Ann Thorac Surg.

[15] Association of American Medical Colleges Group on Diversity and Inclusion.

[16] Brotherton SE, Etzel SI (2018). Graduate medical education, 2017–2018. JAMA.

[17] United States Census Bureau https://www.census.gov/quickfacts/fact/table/US/PST045216.

[18] Betancourt JR, Green AR, Carrillo JE (2003). Defining cultural competence: a practical framework for addressing racial/ethnic disparities in health and health care. Public Health Rep.

[19] Blanchard J, Nayar S, Lurie N (2007). Patient-provider and patient-staff racial concordance and perceptions of mistreatment in the health care setting. J Gen Intern Med.

[20] Richardson LD, Babcock Irvin C, Tamayo-Sarver JH (2003). Racial and ethnic disparities in the clinical practice of emergency medicine. Acad Emerg Med.

[21] The Match: National Resident Matching Program. Results and Data: 2019 Main Residency Match® [PDF File].

